# Cisplatin sensitivity is enhanced in non-small cell lung cancer cells by regulating epithelial-mesenchymal transition through inhibition of eukaryotic translation initiation factor 5A2

**DOI:** 10.1186/1471-2466-14-174

**Published:** 2014-11-07

**Authors:** Guodong Xu, Hui Yu, Xinbao Shi, Lebo Sun, Qingyun Zhou, Dawei Zheng, Huoshun Shi, Ni Li, Xianning Zhang, Guofeng Shao

**Affiliations:** Department of Thoracic & Cardiovascular Surgery, Lihuili Hospital, Ningbo Medical Center, Affiliated Hospital of Medical School of Ningbo University, NO 57 Xingning Road, Ningbo, 315041 China; Department of Pathology, Shanghai Pulmonary Hospital Tongji University School of Medical, Shanghai, 200065 China; Department of Cell Biology and Medical Genetics, Research Center of Molecular Medicine, National Education Base for Basic Medical Sciences, Institute of Cell Biology, Zhejiang University School of Medicine, Hangzhou, Zhejiang Province 310058 China

**Keywords:** N1-guanyl-1, 7-diaminoheptane (GC7), Eukaryotic translation initiation factor 5A2 (eIF5A-2), Epithelial-mesenchymal transition (EMT), Cisplatin, Non-small cell lung cancer (NSCLC)

## Abstract

**Background:**

Epithelial-mesenchymal transition (EMT) has been believed to be related with chemotherapy resistance in non-small cell lung cancer (NSCLC). Recent studies have suggested eIF5A-2 may function as a proliferation-related oncogene in tumorigenic processes.

**Methods:**

We used cell viability assays, western blotting, immunofluorescence, transwell-matrigel invasion assay, wound-healing assay combined with GC7 (a novel eIF5A-2 inhibitor) treatment or siRNA interference to investigate the role of eIF5A-2 playing in NSCLC chemotherapy.

**Results:**

We found low concentrations of GC7 have little effect on NSCLC viability, but could enhance cisplatin cytotoxicity in NSCLC cells. GC7 also could reverse mesenchymal phenotype in NCI-H1299 and prevented A549 cells undergoing EMT after TGF-β1 inducement. eIF5A-2 knockdown resulted in EMT inhibition.

**Conclusion:**

Our data indicated GC7 enhances cisplatin cytotoxicity and prevents the EMT in NSCLC cells by inhibiting eIF5A-2.

**Electronic supplementary material:**

The online version of this article (doi:10.1186/1471-2466-14-174) contains supplementary material, which is available to authorized users.

## Background

Lung cancer is the leading cause of cancer deaths worldwide with non-small cell lung cancer (NSCLC) accounting for approximately 80% of all lung cancer diagnoses [1]. Although surgery is the first choice of treatment, chemotherapy is necessary in most cases in order to improve the therapeutic effect; however, despite many novel chemotherapy regimens and molecular targeted therapies, its pathogenesis is yet to be fully understood, and the prognosis remains poor [2–4].

Epithelial-mesenchymal transition (EMT) is a complex, reversible process which induces epithelial cells to transform to mesenchymal phenotype [5]. These lead to a loss of epithelial characteristics including cell-cell junctions, polarity and epithelial markers, e.g., E-cadherin; and a gain of mesenchymal properties, including stronger migration and invasion capabilities [6] and mesenchymal markers, e.g., vimentin and fibronectin [7]. Although many reports have demonstrated that EMT is involved in drug resistance in NSCLC [8–12], the mechanism is unclear; as such, determining an effective method to inhibit EMT in NSCLC could significantly improve treatment regimes.

Eukaryotic initiation factor (eIF5A) is the the only cellular protein that contains the unusual amino acid hypusine [Ne-(4-amino-2-hydroxybutyl) lysine].It has two isoforms: eIF5A-1 and eIF5A-2. Study demonstrated that accumulating evidence links eIF5A to cell proliferation, cancer progression, invasiveness, metastasis and poor clinical prognosis and the post-translational modifications of eIF-5A could be a suitable target for the potentiation of the activity of anti-cancer agents [13, 14]. eIF5A-2 is located on chromosome 3q26, a region frequently amplified in several types of tumors [15]. It is essential for maintaining cell proliferation [16, 17] and inhibition of eIF5A-2 has been shown to suppress cell proliferation in many tumors [18, 19]. As a result, it has been suggested that eIF5A-2 may function as a proliferation-related oncogene in tumorigenic processes [20].

Several studies have found that N1-guanyl-1,7-diaminoheptane (GC7) suppresses tumor cell proliferation by inhibiting eIF5A-2 [21, 22]. In this study, we aimed to investigate the chemotherapeutic effect of GC7 in NSCLC and determine whether eIF5A-2 mediates EMT and increases chemosensitivity in NSCLC controls.

## Methods

### Cell lines and cell culture

The human NSCLC cell lines, A549 and NCI-H1299, were purchased from the American Type Culture Collection (ATCC; Manassas, VA, USA) and stored following ATCC guidelines. All cells were cultured in Roswell Park Memorial Institute (RPMI) 1640 medium (Invitrogen, Carlsbad, CA, USA) supplemented with 10% fetal bovine serum (FBS; Gibco, Carlsbad, CA, USA) and 1% penicillin-streptomycin (Sigma-Aldrich, St. Louis, MO, USA). The cells were maintained at 37°C in a humidified atmosphere of 5% CO_2_.

### eIF5A-2 siRNA transfection

NSCLC cells were transfected with eIF5A-2 siRNA (10 μmol/mL; Santa Cruz Biotechnology, Dallas, TX, USA) or negative control siRNA (Invitrogen) using Lipofectamine 2000 (Invitrogen) according to the manufacturer’s instructions. The transfection medium was replaced with culture medium 6 h after transfection. All subsequent experiments were performed 24 h after transfection and repeated in triplicate.

### CCK-8 cell viability assay

A Cell Counting Kit-8 (CCK8; Dojindo, Kumamoto, Japan) was used to measure relative cell viability after treatment. NSCLC cells (5 × 10^3^ cells/well) were seeded into 96-well plates and cultured for 24 h. The culture medium was replaced by medium containing the required concentrations of cisplatin or cisplatin combined with GC7, and the cells were incubated for 48 h. CCK-8 solution (10 μL/well) was added, the cells were incubated for a further 4 h, and absorbance was measured at 450 nm using an MRX II microplate reader (Dynex Technologies, Chantilly, VA, USA). Relative cell viability was calculated as a percentage of untreated controls.

### Western blot analysis

The cells were washed twice in ice-cold phosphate buffer solution (PBS) and resuspended in 100 μL cell lysis buffer (Cell Signaling, Danvers, MA, USA) with protease inhibitors (Sigma-Aldrich). The protein concentrations were quantified using a BCA Protein Kit (Thermo Fisher, Rockford, IL, USA). Cell lysates (40 μg/lane) were separated by 10% SDS-PAGE, transferred to polyvinyl diflouride (PVDF) membranes (Millipore, Billerica, MA, USA) and blocked with Tris-buffered saline (TBS) containing 0.1% Tween 20 (TBST) and 5% bovine serum albumin (BSA). The membranes were incubated with anti-E-cadherin, anti-Vimentin (Biovision, Milpitas, CA, USA) or anti-eIF5A-2 (Proteintech, Chicago, IL, USA) antibodies (1:1000) at 4°C overnight, washed three times with TBST and then incubated with the appropriate HRP-conjugated secondary antibodies for 1 h at room temperature. The protein bands were developed by chemiluminescence (GE Healthcare, Piscataway, NJ, USA) and visualized by autoradiography on X-Ray films (Kodak, Rochester, NY, USA). Band densities were estimated using Image-Pro Plus v. 6.0 software (Media Cybernetics, Bethesda, MD, USA) and protein levels were normalized to GAPDH.

### Immunofluorescence

Cells were washed with ice-cold PBS, fixed in 4% paraformaldehyde for 30 min followed by incubation with 3% H_2_O_2_ for 15 min at 37°C and blocked in fetal calf serum for a further 15 min. After incubation with anti-E-cadherin, anti-vimentin or anti-eIF5A-2 antibodies (1:1,000) overnight at 4°C, the cells were washed with ice-cold PBS and incubated for 1 h at room temperature with the appropriate secondary antibodies (1:2000; GE Healthcare): goat anti-mouse FITC-conjugated secondary antibody (E-cadherin) or goat anti-mouse Cy5-conjugated secondary antibody (vimentin). Nuclei were stained with 4,6-diamidino-2-phenylindole (DAPI; Sigma-Aldrich) and the cells were observed by fluorescence confocal microscopy (Olympus, Japan).

### Wound-healing assay

Cells were seeded into six-well plates at a density of 2 × 10^5^ cells/well and cultured with RPMI-1640 medium containing 10% FBS overnight at 37°C in a humidified atmosphere of 5% CO_2_, after which, the medium was changed to RPMI-1640 without FBS and the cells were cultured for a further 24 h until >90% confluence. The cells were harvested by scraping the adherent cells using a plastic 100 μL tip. After transfection with eIF5A-2 siRNA (10 μmol/mL) or treatment with N1-guanyl-1,7-diaminoheptane (GC7; 20 μM) for 6 h, the cells were treated with transforming growth factor-β1 (TGF-β1) at a concentration of 10 ng/mL for 24 h at 37°C in a humidified atmosphere of 5% CO_2_. Micrographs were taken using an inverted phase contrast microscope (Olympus; magnification, 40×) at 0 h and 24 h. The ratio of the remaining wound area relative to the initial wound area was calculated and the wound area was quantified using Image-Pro Plus v. 6.0 software.

### Transwell-matrigel invasion assay

After transfection with eIF5A-2 siRNA (10 μmol/mL) or treatment with GC7 (20 μM) for 6 h, the cells were treated with TGF-β1 (10 ng/mL) for 48 h. The cells were seeded at a density of 5 × 10^4^ cells/well in the upper chamber of a Transwell 24-insert plate with RPMI-1640 medium. The upper chambers were coated with Matrigel (BD Biosciences, San Jose, CA, USA) and the lower chamber contained RPMI-1640 plus 10% FBS medium. After 24 h, the bottom of the inserts were fixed in methanol for 10 min and stained with hematoxylin and eosin (H&E). The cells that had invaded to the lower surface were measured using an inverted phase contrast microscope (Olympus; magnification, 40×) and photographed.

### Statistical analyses

Data were analyzed using GraphPad Prism 5 software (GraphPad, San Diego, CA, USA) using one-way analysis of variance (ANOVA) followed by Tukey post-hoc test. Results are presented as mean ± SEM; *P* <0.05 was considered statistically significant.

## Results

### Low concentrations of GC7 had little cytotoxicity against NSCLC cells

**Figure 1:**
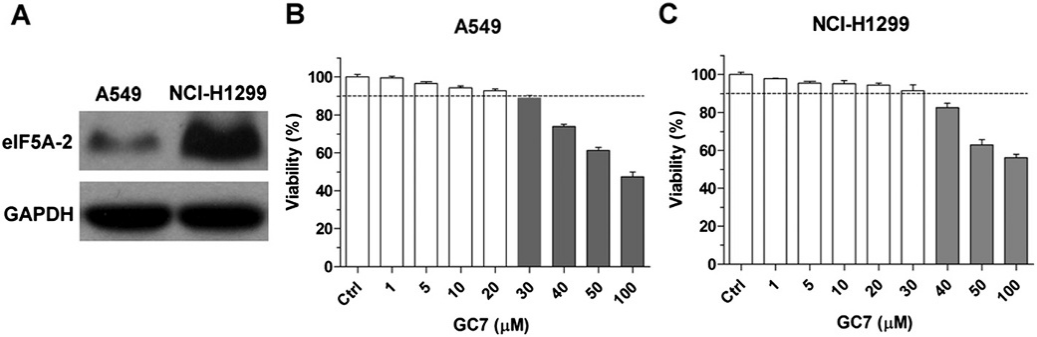
**Low concentrations of GC7 had little cytotoxicity against NSCLC cells. (A)** Western blotting showing eIF5A-2 expression in A549 and NCI-1299 cells. **(B,C)** Low concentrations of GC7 have little effect on cell viability.

Western blot analysis was used to determine eIF5A-2 protein expression in A549 and NCI-H1299 cells. The results showed that eIF5A-2 was expressed in the control cells of both cell lines; however expression was higher in NCI-H1299 cells compared to A549 cells (Figure 1A). In order to test the cytotoxicity of GC7 in A549 and NCI-H1299 NSCLC cell lines CCK-8 cell viability assays were performed. The results showed that GC7 had almost no effect on A549 cell viability between 0 and 20 μM, and NCI-H1299 cell viability was well when GC7 concentrations were less than 30 μM, indicating that GC7 had little cytotoxicity against NSCLC cells at low concentrations (Figure 1B,C). Conversely, at GC7 concentrations exceeding 30 μM in A549 cells or exceeding 40 μM in NCI-H1299 cells, cell viability was significantly inhibited (Figure 1B,C). Some studies have reported that low concentrations of GC7 (10 μM) could inhibit the hypusination of eIF5A2 effectively in some tumor cells [15, 19]. In this case, the 20 μM concentration GC7, which has been showed had little cytotoxicity against NSCLC cells but could inhibit the eIF5A2 activation, was chosen for further co-treatments with cisplatin.

### GC7 enhanced cisplatin sensitivity of mesenchymal NSCLC cells; epithelial NSCLC cells showed greatest sensitivity to cisplatin

**Figure 2:**
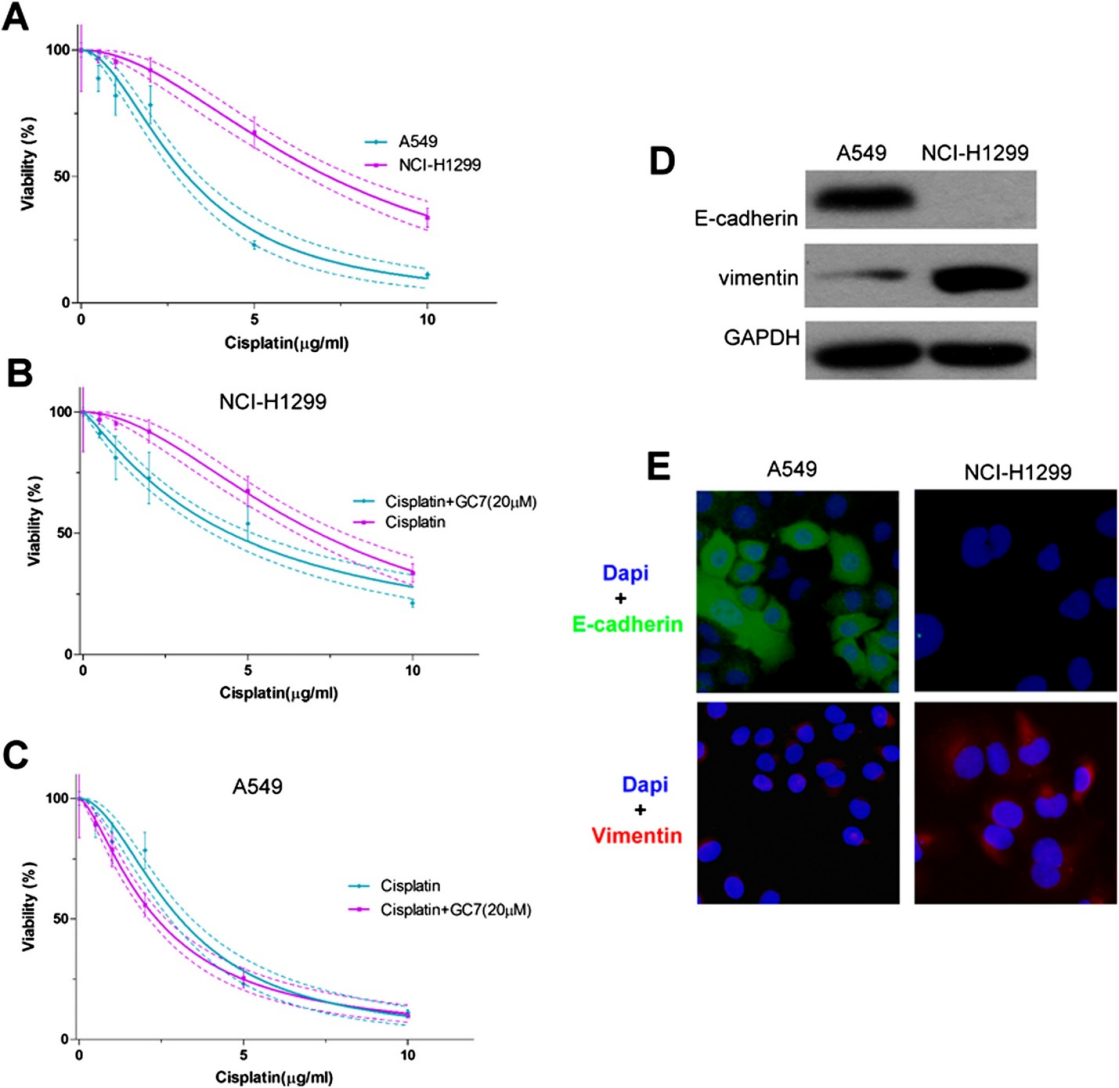
**GC7 enhanced cisplatin sensitivity of mesenchymal NSCLC cells; epithelial NSCLC cells showed greatest sensitivity to cisplatin. (A–C)** GC7 enhances NSCLC cell sensitivity to cisplatin. **(D–E)** A549 and NCI-H1299 express different levels of EMT marker proteins, E-cadherin and vimentin.

CCK-8 assays were carried out to assess the dose-dependence of A549 (epithelial phenotype) and NCI-H1299 (mesenchymal phenotype) cell viability to cisplatin treatment. The results found that increasing doses of cisplatin reduced cell viability in both cell lines (Figure 2A): the IC_50_ values at 72 h were 3.069 μg/mL (2.735–3.402 μg/mL) and 7.140 μg/mL (6.432–7.848 μg/mL) in A549 and NCI-H1299 cells, respectively (Table 1), showing that A549 cells exhibited higher sensitivity to cisplatin than NCI-H1299 cells. When cisplatin was combined with GC7 treatment (20 μM), cisplatin sensitivity increased in both cell lines compared to cisplatin treatment alone: IC_50_ values at 72 h decreased to 4.454 μg/mL (3.848–5.060 μg/mL; *P* <0.0001) in NCI-H1299 (Figure 2B) and 2.360 μg/mL (2.098–2.622 μg/mL; *P* <0.01) in A549 cells (Figure 2C, In Additional file 1), indicating that GC7 increased cisplatin sensitivity most markedly in NCI-H1299 cells.

The difference between phenotypes was examined by western blotting and immunofluorescence to detect expression of E-cadherin (epithelial) and vimentin (mesenchymal) EMT markers in both NSCLC cell lines. The results showed that A549 cells, which were more sensitive to cisplatin, showed higher expression of the epithelial marker E-cadherin, but no expression of the mesenchymal marker vimentin. In contrast, NCI-H1299 showed higher expression of the mesenchymal marker vimentin, but no expression of the epithelial marker E-cadherin (Figure 2D,E).

**Table 1:** **IC**_**50**_ **values for cisplatin in NSCLC cell lines with or without GC7 treatment**

NSCLC cell line	IC _50_(μg/mL) ^▲^	
	Cisplatin	Cisplatin + GC7 (20 μM)
A549	3.069 (2.735–3.402)	2.360 (2.098–2.622)^**^
NCI-H1299	7.140 (6.432–7.848)	4.454 (3.848–5.060)^****^

^▲^IC_50_ concentrations of cisplatin [μg/mL; mean (95% CI)].

^**^ *P* <0.05 vs. cisplatin alone.

^****^ *P* <0.0001 vs. cisplatin alone.

### GC7 enhanced cisplatin sensitivity in NSCLC cells via inhibition of eIF5A-2

**Figure 3:**
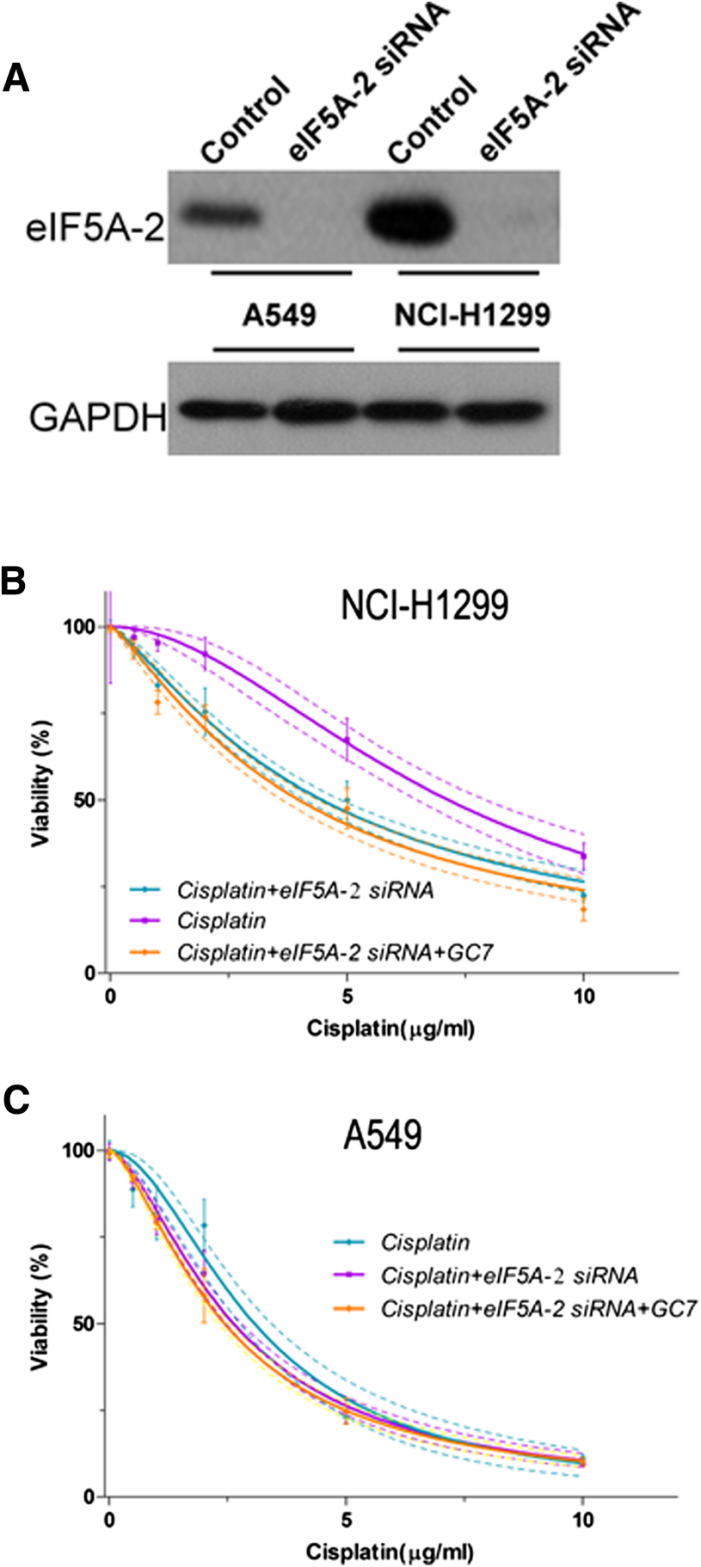
**GC7 enhanced cisplatin sensitivity in NSCLC cells via inhibition of eIF5A-2. (A)** eIF5A-2 siRNA inhibits eIF5A-2 in both A549 and NCI-H1299 cells. **(B–C)** Comparing changes in cisplatin sensitivity in A549 and NCI-H1299 NSCLC cells after treatment with eIF5A-2 siRNA alone or combined with GC7.

GC7 can inhibit the activity of eIF5A-2 (In Additional file 2). In order to discover the mechanism by which GC7 enhanced cisplatin sensitivity, we transfected eIF5A-2 siRNA into A549 and NCI-H1299 cells to interfere with eIF5A-2 expression, and found that eIF5A-2 expression was significantly inhibited in both NSCLC cell lines (Figure 3A). We then treated these transfected cells with cisplatin alone, or cisplatin combined with GC7, and carried out CCK-8 cell viability assays. Without GC7, NCI-H1299 cells were the most sensitive to cisplatin after eIF5A-2 siRNA transfection: the IC_50_ at 72 h was 4.468 μg/mL (4.093–4.842 μg/mL; Table 2). Although A549 cells remained sensitive to cisplatin, the IC_50_ value was lower: 2.626 μg/mL (2.466–2.785 μg/mL; *P* = 0.0145 *vs.* cisplatin alone. Table 2). In contrast, when cisplatin treatment was combined with GC7 after eIF5A-2 siRNA transfection, there was little change in the cisplatin sensitivity of both cell lines: the IC_50_ values at 72 h were 3.982 μg/mL (3.609–4.356 μg/mL; *P* = 0.0648) and 2.434 μg/mL (2.307–2.560 μg/mL; *P* =0.0571) in NCI-H1299 and A549 cells, respectively (Table 2; Figure 3B,C). As GC7 also inhibits eIF5A-1’s activity, we evaluated the role of eIF5A-1 in this process. Western Blot analysis indicated that eIF5A-1 was expressed in the control cells of both cell lines; however the expression of eIF5A-1 was higher in NCI-H1299 cells compared to A549 cells. Moreover, We also evaluated the effect of eIF5A-1 in the siRNA transfected cell. The results showed that when cisplatin treatment was combined with GC7 after eIF5A-1 siRNA transfection, there was little change in the cisplatin sensitivity of both cell lines (In Additional file 3).

**Table 2:** **IC**_**50**_ **values for cisplatin in NSCLC lines with or without GC7 treatment after eIF5A-2 inhibition**

NSCLC cell line	IC _50_(μg/mL) ^▲^	
	siRNA + Cisplatin	siRNA + Cisplatin + GC7 (20 μM)
A549	2.626 (2.466–2.785)	2.434 (2.307–2.560)
NCI-H1299	4.468 (4.093–4.842)	3.982 (3.609–4.356)

^▲^IC_50_ values indicate the cisplatin concentration [μg/mL; mean (95% CI)].

### GC7 regulated EMT in NSCLC cells via inhibition of eIF5A-2

**Figure 4:**
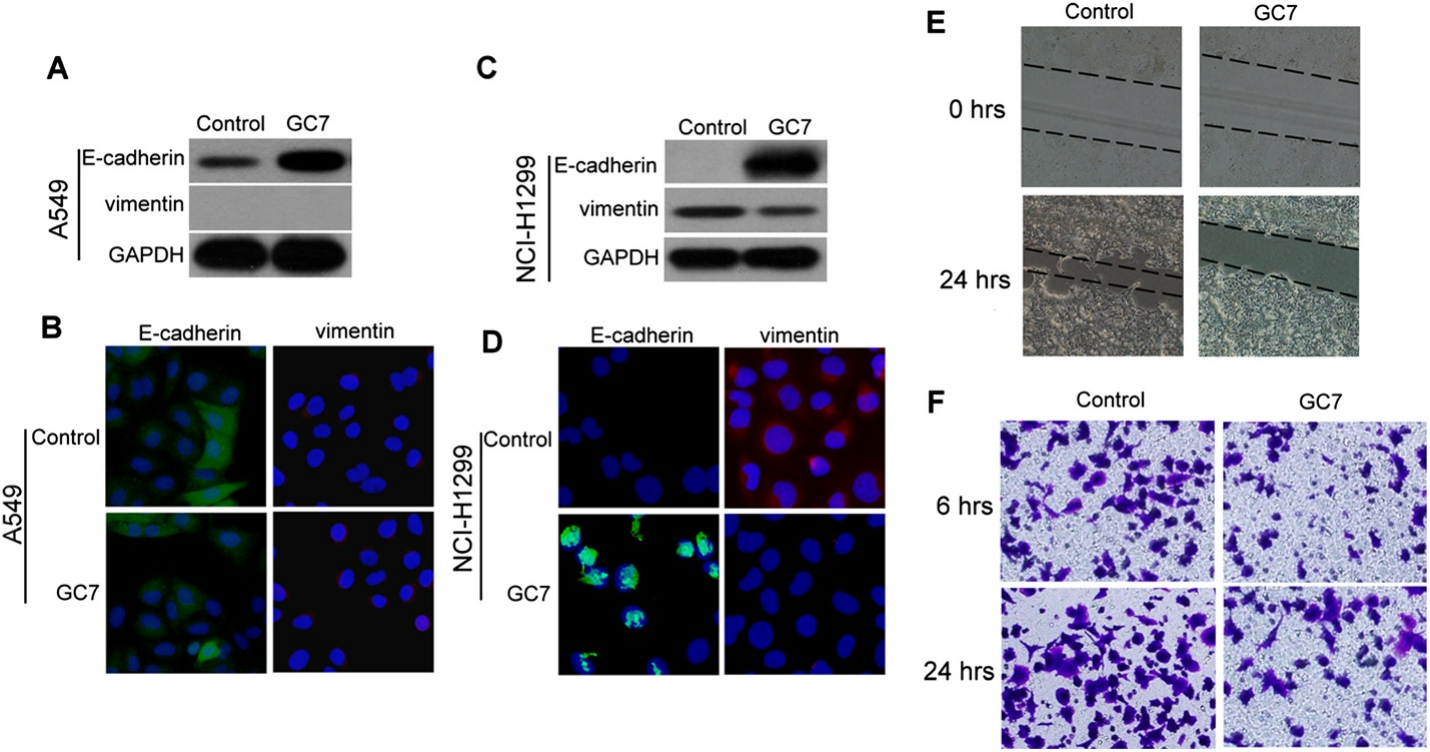
**GC7 regulated EMT in NSCLC cells via inhibition of eIF5A-2. (A–B)** Western blotting and immunofluorescence showed no significant changes in expression levels of EMT marker proteins, E-cadherin and vimentin, are observed in A549 cells after GC7 treatmen. **(C-D)** Western blotting and immunofluorescence showed significant changes in expression levels of EMT marker proteins, E-cadherin and vimentin, are observed in NCI-H1299 cells after GC7 treatment. **(E)** The migration and **(F)** invasion capabilities are weaker in NCI-H1299 cells after GC7 treatment.

Having established that GC7 enhanced the chemotherapeutic effect of cisplatin in NCI-H1299 more than in A549 cells, we wished to determine whether the mechanism was related to EMT. After GC7 treatment for 72 h, A549 cells retained their epithelial characteristics (Figure 4A,B), whereas NCI-H1299 cells displayed a loss of mesenchymal properties and a gain of epithelial properties, appearing a reduction in their migration and invasion capabilities (Figure 4E,F). Furthermore, the NCI-H1299 cells showed increased levels of epithelial marker E-cadherin and lower levels of mesenchymal marker vimentin (Figure 4C,D).

**Figure 5:**
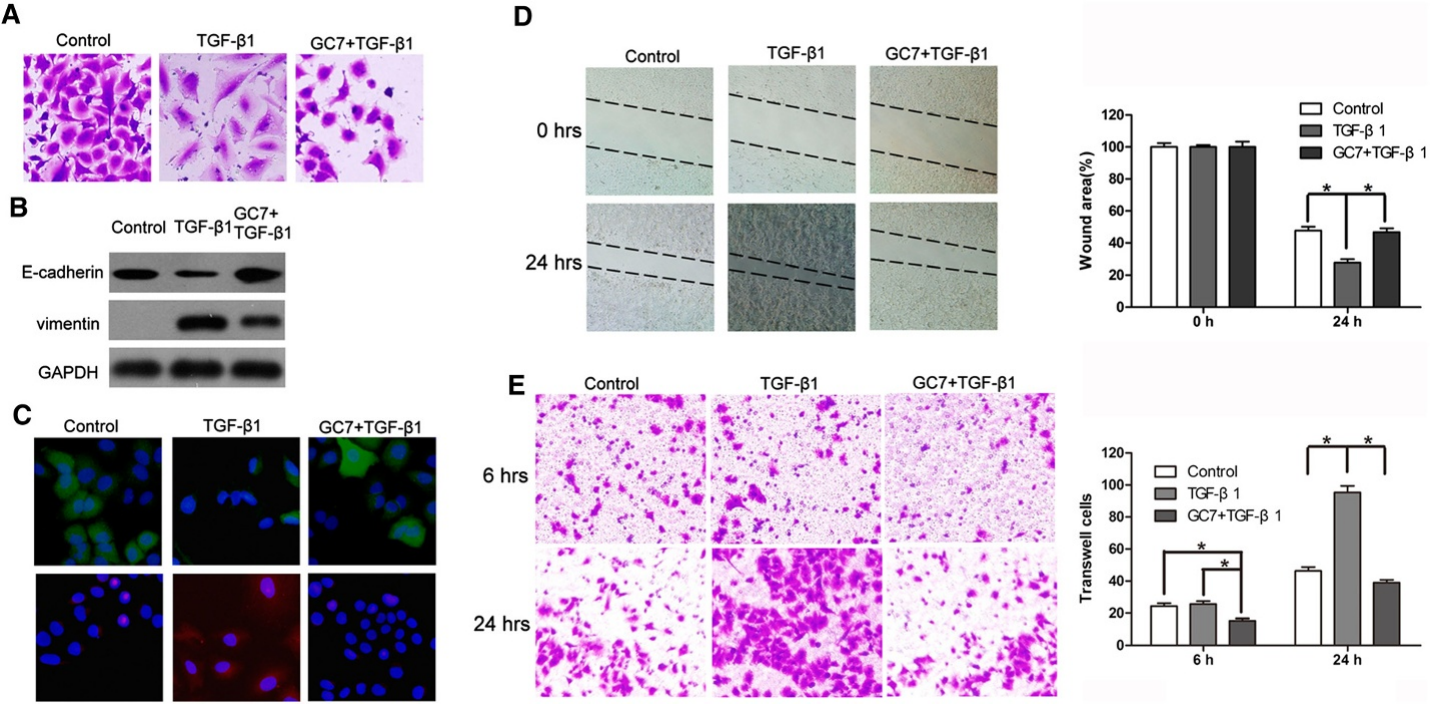
**GC7 pre-treatment prevents A549 cells from undergoing EMT after TGF-β1 stimulation. (A)** morphology **(B)** Western blotting **(C)** immunofluorescence **(D)** wound-healing assay **(E)** Transwell-Matrigel invasion assay.

Several reports have shown that TGF-β1 could induce epithelial NSCLC cells to undergo EMT. In this study, TGF-β1 exposure (10 ng/mL for 48 h) transformed epithelial A549 cells to mesenchymal phenotype, causing the cells to develop an elongated appearance, irregular pseudopodia, weaker cell-cell junctions (Figure 5A) and stronger migration and invasion capabilities compared to control cells (Figure 5D,E). In addition, the cells showed lower levels of epithelial marker E-cadherin and increased levels of mesenchymal marker vimentin (Figure 5B,C). Conversely, if the A549 cells were pre-treated with GC7 before exposure to TGF-β1, the cells retained their epithelial appearance, levels of EMT markers, and migration and invasion capabilities (Figure 5A–E).

**Figure 6:**
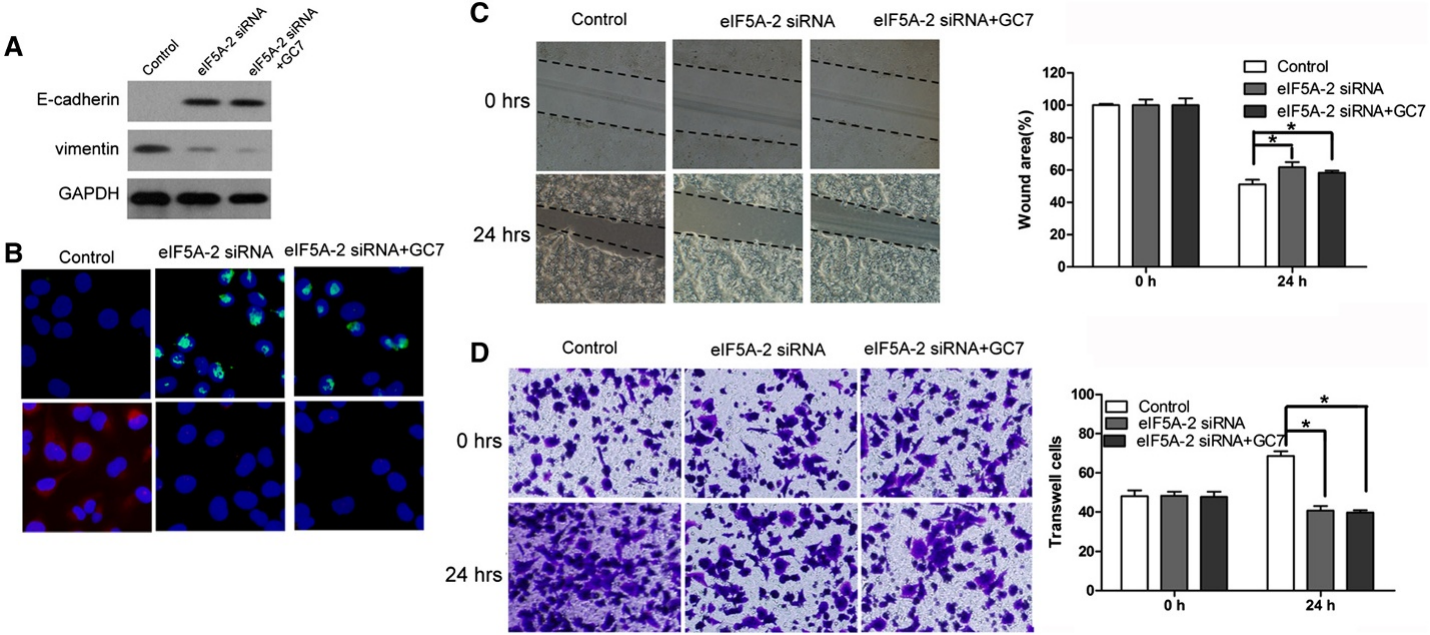
**GC7 reverses EMT in NCI-H1299 cells via eIF5A-2 regulation. (A)** Western blotting **(B)** immunofluorescence **(C)** wound-healing assay **(D)** Transwell-Matrigel invasion assay.

In order to verify whether eIF5A-2 was a key factor in GC7 regulation of EMT, we transfected eIF5A-2 siRNA into NCI-H1299 cells without carrying out GC7 treatment. The results showed that the transfected NCI-H1299 cells transformed from mesenchymal phenotype to epithelial phenotype (Figure 6A–D). Conversely, when the transfected cells were treated with GC7, the cells stayed as epithelial phenotype (Figure 6A–D).

## Discussion

EIF5A-2 is a member of the eukaryotic initiation factor family. It is located on chromosome 3q26, a region frequently amplified in several tumors, and is highly expressed in tumors such as colorectal cancer [23], ovarian cancer [24] and bladder cancer [25]. Overexpression of eIF5A-2 has been reported to enhance invasion and metastasis in malignancies [20, 26], for example, He et al. reported that overexpression of eIF5A-2 was correlated with invasion in NSCLC and was a poor prognostic marker of NSCLC [20]. In addition, eIF5A has been shown to induce EMT in hepatocellular carcinoma [26] and colorectal carcinoma [27].

Many studies have shown that EMT is related to carcinogenicity, metastasis and poor prognosis in many tumors including NSCLC [28–31], and it has been suggested that EMT is involved in drug resistance in NSCLC [10–12]. During EMT, epithelial markers such as E-cadherin decrease, while mesenchymal markers such as vimentin increase [8]. In our study, we showed that NCI-H1299 cells, a mesenchymal phenotype, expressed higher levels of eIF5A-2. In contrast A549 cells, an epithelial phenotype, expressed lower levels of eIF5A-2. Furthermore, we showed that epithelial A549 cells were more sensitive to cisplatin, whereas the mesenchymal NCI-H1299 cells were related to drug resistance.

Several studies have reported that GC7 possesses antitumor properties [32, 33] and significantly suppresses tumor cell proliferation [21, 22]. The enzymes deoxyhypusine synthase (DHS) and deoxyhypusine hydroxylase (DOHH) are required to catalyze the post-translational modifications which lead to the activation of eIF5A2 [33]. GC7 is a potent inhibitor of DHS, thereby inducing eIF5A-2 inactivation. Our study found that mesenchymal NCI-H1299 cells changed to epithelial phenotype when co-treated with GC7; furthermore, in agreement with other reports, we found that GC7 not only increased NCI-H1299 sensitivity to cisplatin cells but also reduced the migration and invasion capabilities of NCI-H1299 cells.

TGF-β signaling plays an important role in the EMT process through regulation of Snail, SOX2, SOX4 and ID1 [34–36] and has been reported to stimulate NSCLC cells to undergo EMT [29, 37, 38]. In previous study we found that after exposure to TGF-β1, epithelial A549 cells changed to mesenchymal phenotype, developing a mesenchymal appearance, higher levels of vimentin, lower levels of E-cadherin and stronger migration and invasion capabilities [39]. In this study, we mainly investigate GC7 whether can be reversed this effect, and the result showed that EMT could be prevented if A549 cells were pre-treated with GC7. This suggested that eIF5A-2 might be an upstream factor regulating EMT and thereby plays an important role in EMT phenotype changes.

## Conclusion

In conclusion, our study found that GC7 changed NCI-H1299 cells from mesenchymal phenotype to epithelial phenotype and enhanced their sensitivity to cisplatin via inhibition of eIF5A-2, whereas GC7 prevented epithelial A549 cells from undergoing EMT changes via inhibition of eIF5A-2. This suggests that eIF5A-2 may be a key regulatory factor in EMT and drug resistance in NSCLC. As such, inhibition of eIF5A-2 could enhance NSCLC sensitivity to chemotherapeutics, prevent or reverse EMT, and reduce the migration and invasion capabilities of NSCLC cells. These findings not only support the use of EIF5A2 as an adverse prognostic marker in NSCLC patients, but may also offer a novel approach for the treatment of NSCLC.

## Electronic supplementary material

Additional file 1: Figure S1: Evaluate the possible synergism between GC7 and cisplatin on the growth inhibition of NSCLC (A) NCI-H1299 (B) A549. (TIF 159 KB)

Additional file 2: Figure S2: Fluorogram of SDS-PAGE separated hypusinated--eIF5A1/eIF5A2 protein (Hypusined eIF5A isoform) in NCI-H1299 and A549 cells protein lysates after 48 h incubation with or without GC7 (20 μM) in the presence of [1,8-3H]-spermidine. (TIF 81 KB)

Additional file 3: Figure S3: (A-B) Comparing changes in cisplatin sensitivity in A549 and NCI-H1299 NSCLC cells after treatment with eIF5A-1 siRNA alone or combined with GC7. (C) Western blotting showing eIF5A-1 expression in A549 and NCI-1299 cells. (D) eIF5A-1 siRNA inhibits eIF5A-2 in both A549 and NCI-H1299 cells. (E) The effects of GC7 and cisplatin on the expression of the two isoforms of eIF-5A in the NSCLC cell lines. (TIF 6 MB)
