# Supplementary figures and images for: Cisplatin sensitivity is enhanced in non-small cell lung cancer cells by regulating epithelial-mesenchymal transition through inhibition of eukaryotic translation initiation factor 5A2
Source: BMC Pulm Med. 2014 Nov 7;14:174. doi: 10.1186/1471-2466-14-174 (PMC4232729; doi:10.1186/1471-2466-14-174)

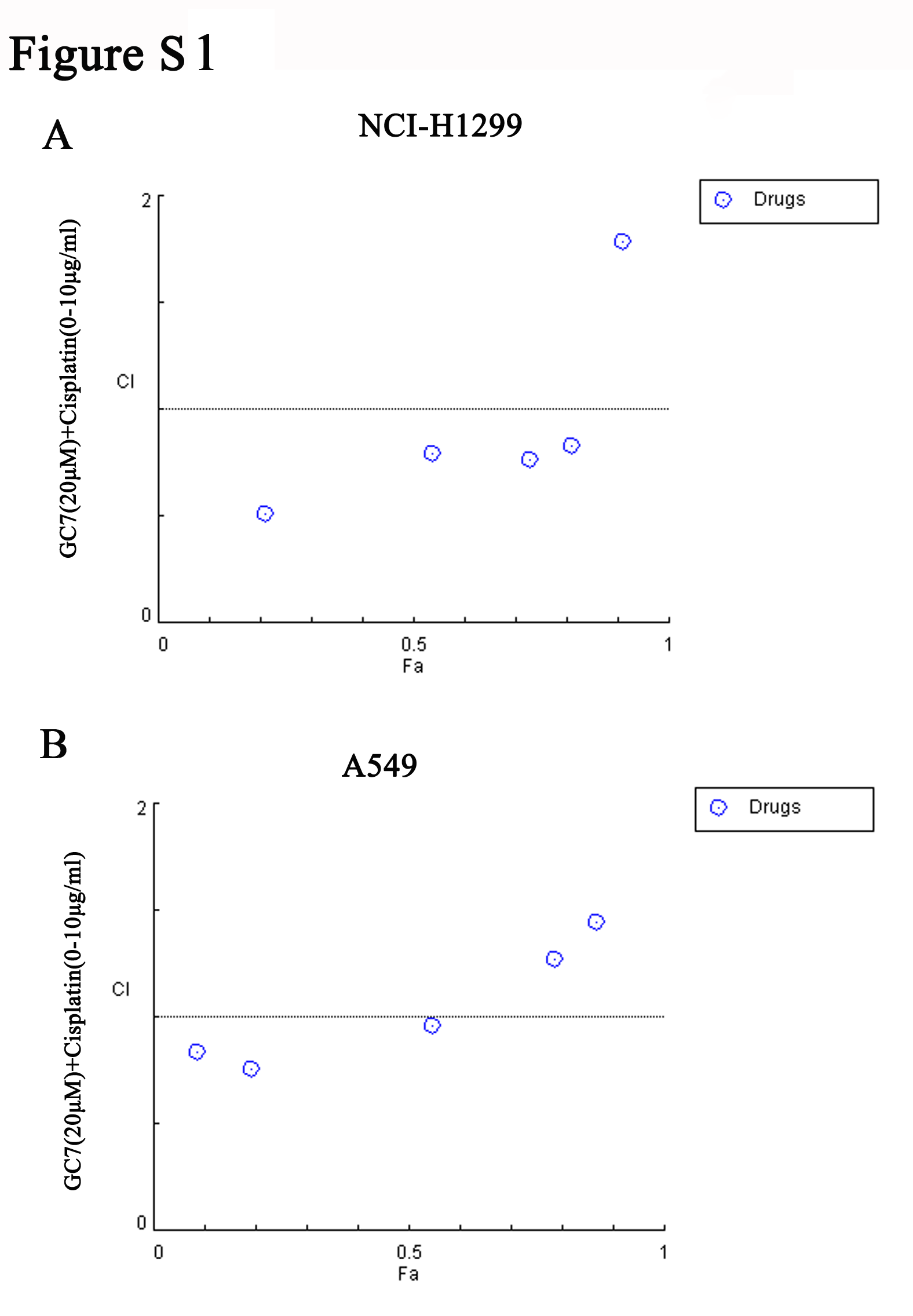

Supplement: Supplementary file 1 — Additional file 1: Figure S1: Evaluate the possible synergism between GC7 and cisplatin on the growth inhibition of NSCLC (A) NCI-H1299 (B) A549. (TIF 159 KB) [file 12890_2013_609_MOESM1_ESM.tif]

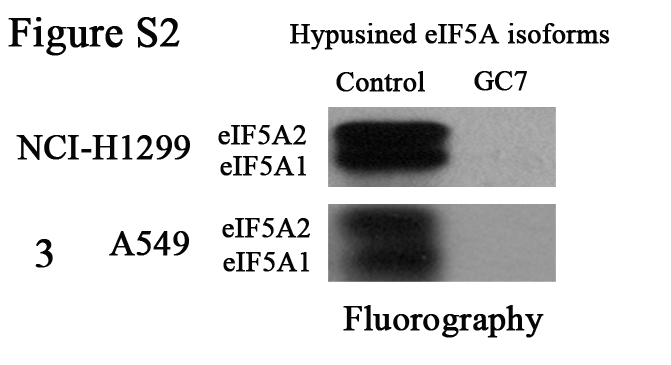

Supplement: Supplementary file 2 — Additional file 2: Figure S2: Fluorogram of SDS-PAGE separated hypusinated--eIF5A1/eIF5A2 protein (Hypusined eIF5A isoform) in NCI-H1299 and A549 cells protein lysates after 48 h incubation with or without GC7 (20 μM) in the presence of [1,8-3H]-spermidine. (TIF 81 KB) [file 12890_2013_609_MOESM2_ESM.tif]

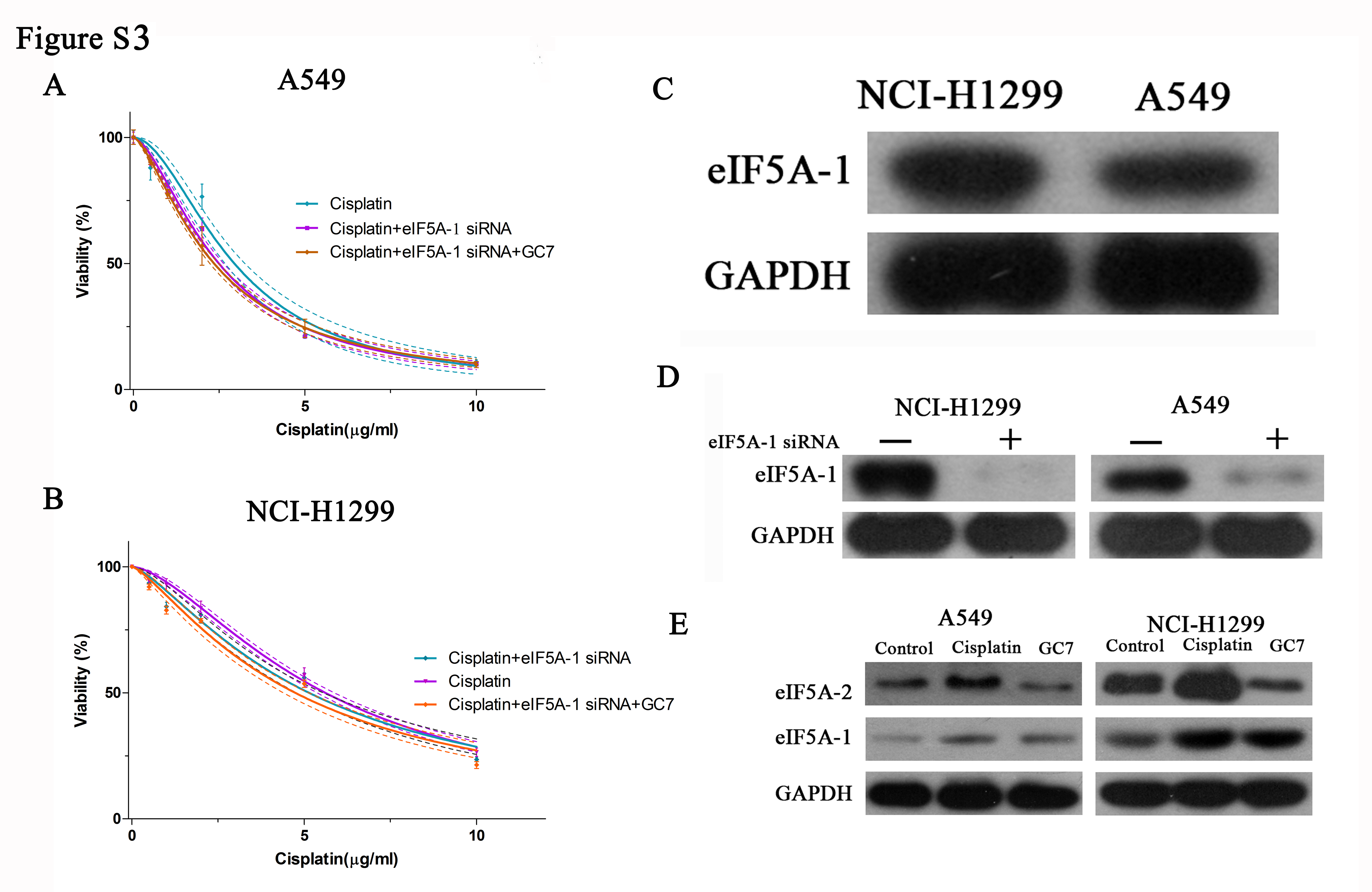

Supplement: Supplementary file 3 — Additional file 3: Figure S3: (A-B) Comparing changes in cisplatin sensitivity in A549 and NCI-H1299 NSCLC cells after treatment with eIF5A-1 siRNA alone or combined with GC7. (C) Western blotting showing eIF5A-1 expression in A549 and NCI-1299 cells. (D) eIF5A-1 siRNA inhibits eIF5A-2 in both A549 and NCI-H1299 cells. (E) The effects of GC7 and cisplatin on the expression of the two isoforms of eIF-5A in the NSCLC cell lines. (TIF 6 MB) [file 12890_2013_609_MOESM3_ESM.tif]
